# Therapeutic Potential of Adipose-Derived Stem Cell-Conditioned Medium and Extracellular Vesicles in an In Vitro Radiation-Induced Skin Injury Model

**DOI:** 10.3390/ijms242417214

**Published:** 2023-12-07

**Authors:** Zhixiang Lin, Yoichiro Shibuya, Yukiko Imai, Junya Oshima, Masahiro Sasaki, Kaoru Sasaki, Yukiko Aihara, Vuong Cat Khanh, Mitsuru Sekido

**Affiliations:** 1 Department of Plastic and Reconstructive Surgery, Institute of Medicine, University of Tsukuba, Tsukuba 305-8575, Ibaraki, Japanimai.yukiko.cj@ms.hosp.tsukuba.ac.jp (Y.I.); sasakku0714@gmail.com (M.S.);; 2 Department of Plastic and Reconstructive Surgery, Mito Saiseikai General Hospital, Mito 311-4145, Ibaraki, Japan; 3 Laboratory of Regenerative Medicine and Stem Cell Biology, University of Tsukuba, Tsukuba 305-8575, Ibaraki, Japan

**Keywords:** adipose-derived stem cells, extracellular vesicles, fibroblasts, radiation, radiotherapy

## Abstract

Radiotherapy (RT) is one of three major treatments for malignant tumors, and one of its most common side effects is skin and soft tissue injury. However, the treatment of these remains challenging. Several studies have shown that mesenchymal stem cell (MSC) treatment enhances skin wound healing. In this study, we extracted human dermal fibroblasts (HDFs) and adipose-derived stem cells (ADSCs) from patients and generated an in vitro radiation-induced skin injury model with HDFs to verify the effect of conditioned medium derived from adipose-derived stem cells (ADSC-CM) and extracellular vesicles derived from adipose-derived stem cells (ADSC-EVs) on the healing of radiation-induced skin injury. The results showed that collagen synthesis was significantly increased in wounds treated with ADSC-CM or ADSC-EVs compared with the control group, which promoted the expression of collagen-related genes and suppressed the expression of inflammation-related genes. These findings indicated that treatment with ADSC-CM or ADSC-EVs suppressed inflammation and promoted extracellular matrix deposition; treatment with ADSC-EVs also promoted fibroblast proliferation. In conclusion, these results demonstrate the effectiveness of ADSC-CM and ADSC-EVs in the healing of radiation-induced skin injury.

## 1. Introduction

Radiotherapy (RT) has become an important therapeutic option in curative and palliative oncological treatment regimens for a variety of malignant tumors. More than 1.5 million patients are diagnosed with new cases of malignant tumors each year [1], and more than 50% of patients with malignant tumors undergo RT [2,3]. RT is based on the principle that it causes damage to cellular organelles and produces direct and indirect DNA single-strand breaks and/or double-strand breaks, inducing cell cycle arrest, senescence, and multiple modes of cell death, including apoptosis, necrosis, autophagy, and mitotic catastrophe [4]. RT indirectly induces DNA lesions through the formation of reactive oxygen species and reactive nitrogen species. The most critical and hard-to-repair types of DNA damage are labeled through phosphorylation of histone H2AX on serine 139 (γH2AX). This, in turn, recruits all the components of the machinery involved in the DNA damage response, which results in cell cycle arrest and DNA repair. Severe and massive DNA lesions lead to cell death, especially in cancer cells, which repair DNA damage less efficiently than healthy cells [5]. Previous studies have shown that RT promotes lipid oxidation and ferroptosis [6], while biochemical studies have demonstrated that multilayered liposomes treated with therapeutically relevant doses of radiation therapy undergo peroxidation and lipid fragmentation, leading to model membrane rupture [7]. The absorption of RT by water leads to the formation of oxygen radicals, which subsequently attack polyunsaturated fatty acids and cause lipid peroxidation. The consequence of excessive lipid oxidation is ferroptosis. RT has been shown to increase lipid oxidation in a dose-dependent manner [6].

Despite advances in radiation therapy technology, most patients still suffer from radiation-induced side effects. Over 95% of patients undergoing RT may develop some form of radiation dermatitis or radiation-induced skin injury [8], which are commonly graded as acute, consequential-late, or chronic. Acute changes occur within 90 days [9]. The earliest visible skin reaction is erythema, which may later evolve into desquamation or even ulceration [10]. This condition can affect a patient’s quality of life during and after treatment. If severe, there is a risk of limiting the radiation dose or interrupting the treatment plan, which may negatively affect the treatment outcome. Alternatively, the skin may appear relatively normal for months to years after RT when chronic radiation-induced skin injury occurs [9]. Chronic radiation-induced skin injury is a permanent, progressive, and irreversible form of dermatitis that severely affects a patient’s quality of life [11]. Thus, managing radiation-induced skin injury during and after treatment is an important aspect of cancer care.

More specifically, an inflammatory response occurs in the skin immediately after radiation exposure, whereby neutrophils are attracted to the irradiated site by cytokines released from damaged skin and endothelial cells, and pro-inflammatory cytokines such as interleukin-6 (*IL6*) are released, which perpetuates this inflammation [12]. These factors lead to self-perpetuating tissue damage and loss of protective barriers [13]. Wound healing is impaired because of the destruction of basal keratinocytes, and thus repeated exposure does not allow any time for tissue or cell repair. Each additional exposure to RT leads to further direct tissue damage, inflammation, and impaired epithelial regeneration, all of which contribute to the development of acute radiation injury [14].

A variety of interventions are currently being used to treat radiation-induced skin injury, and patients are often advised to use skin emollients or moisturizers throughout the RT process. One of the most common of these is a water-based cream, which is used to both prevent and treat erythema and dry flaking [15]. Aloe vera is also used at some centers, although some studies have shown no benefit from its use [16]. Given their anti-inflammatory properties, topical corticosteroids have attracted interest in the treatment of radiation-induced skin injury, and a number of small clinical trials have evaluated the efficacy of topical corticosteroids in reducing the frequency and severity of radiation-induced skin injury, with inconsistent results [9]. Some studies have shown no statistically significant difference between steroids (0.1% mometasone furoate cream [17]; 0.2% hydrocortisone valerate [18]) and placebos, whereas other groups have shown a reduction in the severity or frequency of acute radiation-induced skin injury in the topical steroid group [19,20,21]. The results have been mixed; therefore, the search for new clinically feasible solutions to the problem of radiation-induced skin injury wound healing continues.

It has been shown that mesenchymal stem cell (MSC) treatment enhances the survival and migration of fibroblasts and increases the extracellular matrix (ECM) deposition of fibroblasts, thereby enhancing skin wound healing [22]. Adipose-derived stem cells (ADSCs), a type of MSC that is abundant in adipose tissue, have recently attracted attention as a source of MSCs because of their ease of collection and similar characteristics to MSCs. Numerous studies have shown that the functions of MSCs are mainly directed through paracrine pathways of soluble mediators such as growth factors, cytokines, chemokines, and extracellular vesicles (EVs) [23]. EVs are a heterogeneous group of membrane vesicles that include exosomes and microvesicles, which are released from MSCs and contain various molecules such as messenger RNA (mRNA), microRNA (miRNA), proteins, and lipids [24]. Many studies have shown that MSC-derived EVs (MSC-EVs) have similar biological functions to MSCs themselves and can be used as a possible therapeutic approach [25]. EVs of ADSCs (ADSC-EVs) modulate inflammatory responses, accelerate angiogenesis, increase the migration and proliferation of keratinocytes and fibroblasts, and activate collagen and elastin synthesis in fibroblasts to enhance wound healing [26,27]. However, the potential role of conditioned medium from ADSCs (ADSC-CM) and ADSC-EVs on radiation-induced skin injury and the exact mechanism of their influence on healing remain unknown. We hypothesize that they enhance healing by modulating the inflammatory response, reducing human dermal fibroblast (HDF) apoptosis, and promoting fibroblast proliferation and ECM deposition.

In this study, we examined and analyzed ADSCs and ADSC-EVs, characterized ADSC-EVs, and observed the effects of ADSC-CM and ADSC-EVs on the proliferation, apoptosis, collagen synthesis, and gene expression of radiation-irradiated HDFs.

## 2. Results

### 2.1. Characterization of ADSCs and ADSC-EVs

**Figure 1:**
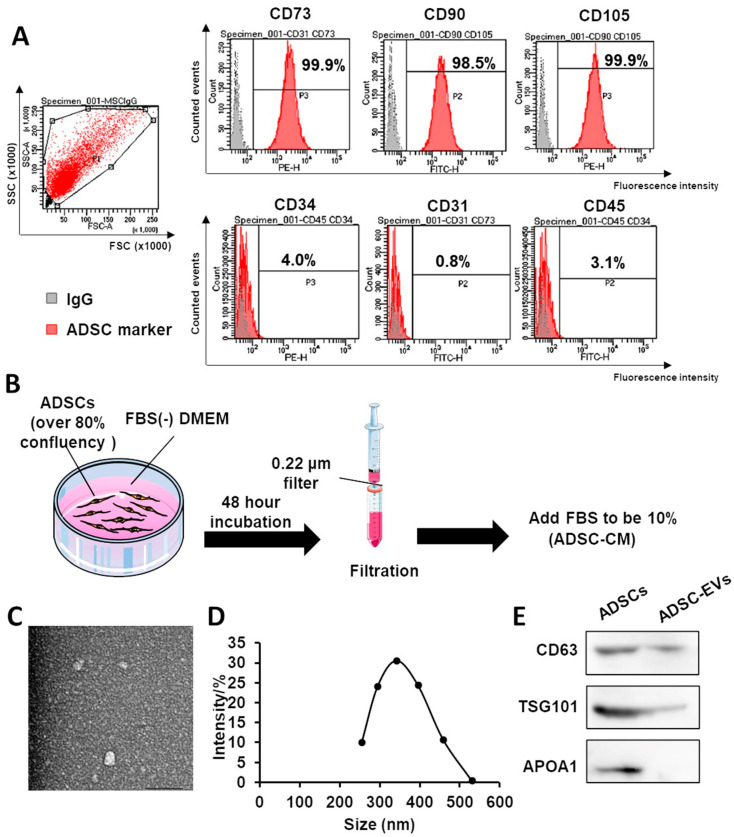
Preparation of ADSC-CM and characterization of ADSCs and ADSC-EVs. (**A**) Flow cytometry analysis showed that ADSCs were highly positive for CD73, CD90, and CD105 but negative for CD34, CD31, and CD45. (**B**) Schematic representation of ADSC-CM preparation. (**C**) Representative image showing the morphology of ADSC-EVs obtained with TEM (scale bar = 100 nm). (**D**) Size distribution profile of ADSC-EVs by DLS. (**E**) The expression of the ADSC-EV markers CD63 and TSG101 was confirmed with Western blot analysis. Abbreviations: ADSCs, adipose-derived stem cells; DMEM, Dulbecco’s modified Eagle’s medium; FBS, fetal bovine serum; ADSC-CM, conditioned medium of adipose-derived stem cells; ADSC-EVs, extracellular vesicles of adipose-derived stem cells; DLS, dynamic light scattering; TEM, transmission electron microscopy.

The flow cytometry analysis showed that ADSCs were highly positive for CD73, CD90, and CD105 but negative for CD34, CD31, and CD45 (Figure 1A). ADSC-EVs were isolated from the ADSC-CM. The preparation method for ADSC-CM is shown in Figure 1B. The cup-shaped morphology of the ADSC-EVs was observed using transmission electron microscopy (TEM) (Figure 1C). The size of the ADSC-EVs was measured using dynamic light scattering (DLS; Zetasizer Nano ZS, Malvern Instruments, Malvern, UK). ADSC-EVs were smaller than 1000 nm (Figure 1D). Western blotting showed that the ADSC-EV markers CD63 and TSG101 were expressed in ADSC-EVs. ADSCs were used as a positive control group and APO1A1 was used as a negative marker of ADSC-EV (Figure 1E).

### 2.2. Construction of the In Vitro Radiation-Induced Skin Injury Model

**Figure 2:**
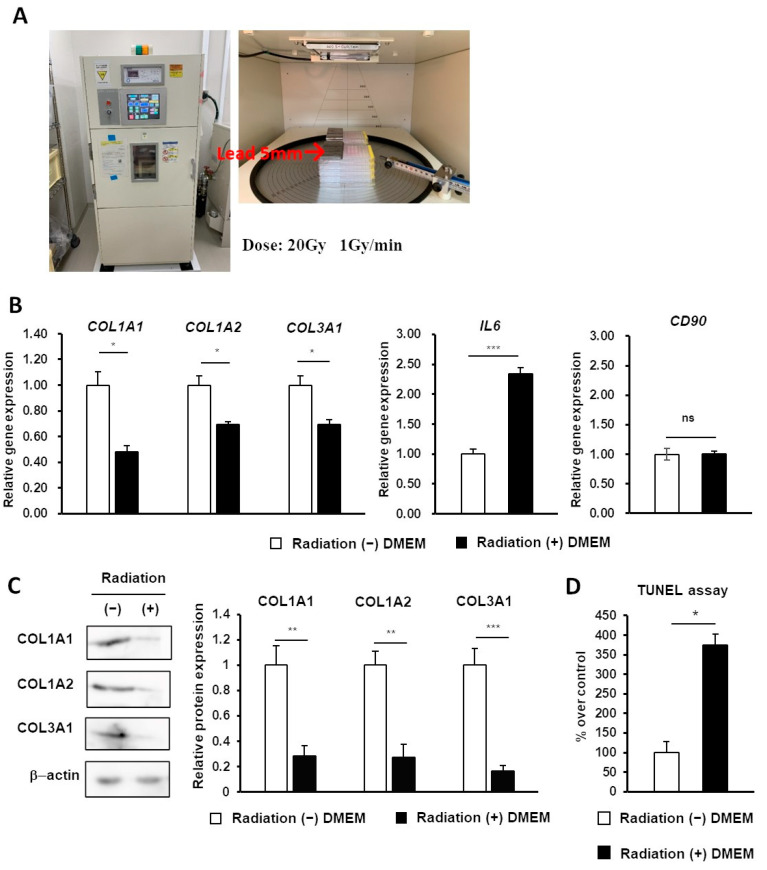
Construction of the radiation-induced skin injury model. (**A**) Schematic representation of radiation exposure. Radiation was administered as a single dose of 1 Gy per minute over 20 min (total dose of 20 Gy). The radiation-negative group was covered with a 5 mm thick lead plate. (**B**) qPCR analysis was performed on the radiation-exposed group compared with the non-radiation-exposed control group to examine the radiation-induced skin injury model established with HDFs. (**C**) Western blotting of collagen proteins was performed on the radiation-exposed group compared with the non-radiation-exposed control group. (**D**) A TUNEL assay was performed on the radiation-exposed group compared with the non-radiation-exposed control group to evaluate radiation-induced apoptosis. Statistical analyses were performed to compare each group of samples individually (* *p* < 0.05, ** *p* < 0.01, *** *p* < 0.001; ns, no statistical significance) (*n* = 3). Abbreviations: radiation (−), non-radiation group; radiation (+), radiation group; qPCR, quantitative real-time polymerase chain reaction; HDFs, human dermal fibroblasts.

HDFs were cultured as a monolayer from skin tissue collected from patients and were cultured until they reached over 80% confluency. Radiation exposure was performed 1 h before the addition of reagents for each experimental group (Figure 2A). The expression of collagen genes *COL1A1*, *COL1A2*, *COL3A1*, the inflammatory marker *IL6*, and the stem cell marker *CD90* were compared using quantitative real-time polymerase chain reaction (qPCR) to evaluate the radiation-induced skin injury model established with HDFs. The results showed that *COL1A1*, *COL1A2*, and *COL3A1* expression was significantly decreased compared with the non-irradiation group, *IL6* expression was significantly increased compared with the non-irradiation group, and *CD90* expression was not statistically significant compared with the non-irradiation group (Figure 2B). In addition to gene expression, the expression of collagen proteins COL1A1, COL1A2, and COL3A1 were compared using Western blotting. As a result, COL1A1, COL1A2, and COL3A1 protein expressions were significantly impaired in the radiation group compared with the non-irradiation group (Figure 2C). Moreover, a TUNEL assay was performed to evaluate radiation-induced cytotoxicity. The number of apoptotic cells was significantly increased compared with the non-irradiation group (Figure 2D). These data suggested that the model can be used in studies of radiation-induced skin injury.

### 2.3. Effects of ADSC-CM and ADSC-EVs on HDFs

**Figure 3:**
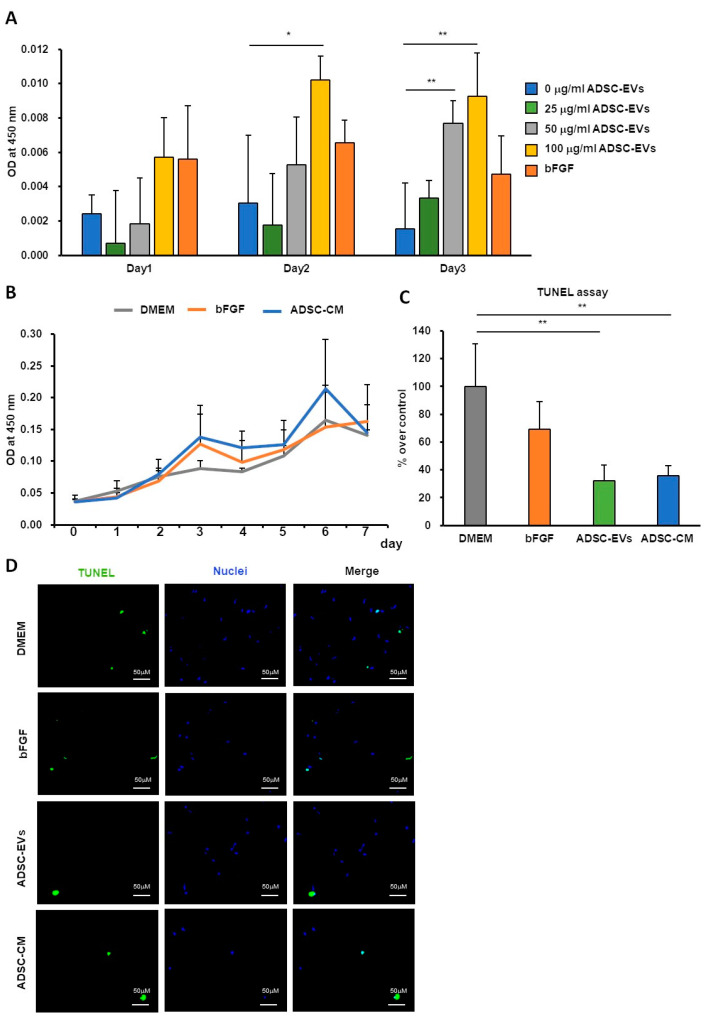
Effects of ADSC-CM and ADSC-EVs on HDFs. (**A**) The proliferation of HDFs treated with different concentrations of ADSC-EVs was evaluated using a WST-1 assay (*n* = 4). (**B**) The proliferation of HDFs treated with ADSC-CM was evaluated using a WST-1 assay (*n* = 5). (**C**) The number of apoptotic HDFs treated with ADSC-EVs or ADSC-CM was evaluated using a TUNEL assay (*n* = 4). (**D**) Representative micrographs of apoptotic cells after 48 h of ADSC-EVs or ADSC-CM treatment. Green: apoptotic cells; blue: nuclei stained with DAPI. Scar bar: 50 µm. Statistical analyses compared samples on the same day (* *p* < 0.05, ** *p* < 0.01). Abbreviations: bFGF, basic fibroblast growth factor.

The proliferation of HDFs cultured for 1, 2, and 3 days in a medium containing ADSC-EVs is shown in Figure 3A. ADSC-EVs promoted the proliferation of HDFs in a dose-dependent manner. ADSC-CM did not significantly promote the proliferation of HDFs but also did not inhibit the proliferation of HDFs (Figure 3B). However, ADSC-CM- or ADSC-EV-treated HDFs significantly reduced the number of apoptotic cells (Figure 3C,D and Supplementary Figure S1).

### 2.4. ADSC-CM and ADSC-EVs Promoted Collagen Synthesis of HDFs

**Figure 4:**
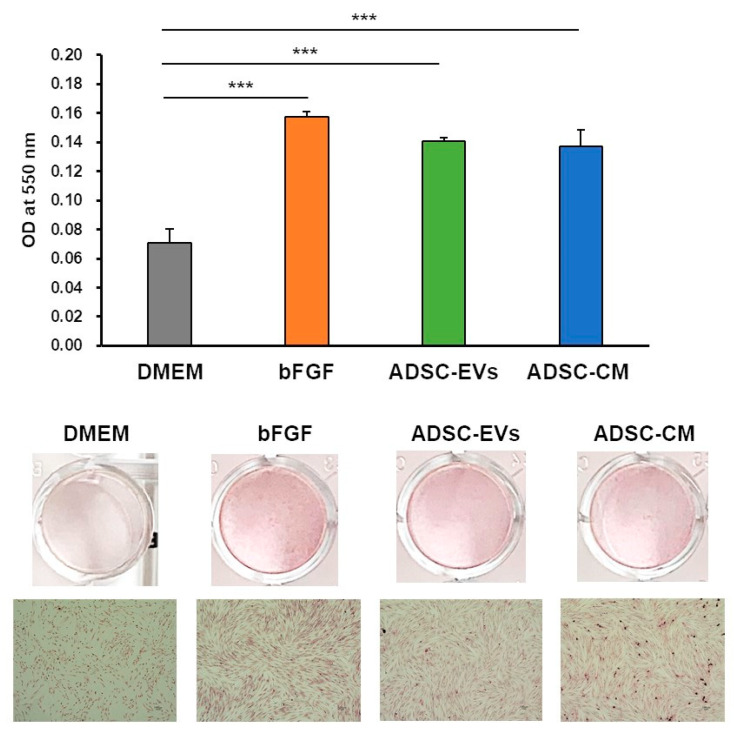
The effects of collagen synthesis in HDFs treated with ADSC-CM or ADSC-EVs were analyzed using picrosirius red staining. Statistical analyses were performed to compare each sample with the control group DMEM (*** *p* < 0.001) (*n* = 3) (scale bar = 100 µm).

In this study, collagen synthesis was evaluated in HDFs treated with ADSC-EVs or ADSC-CM. Ascorbic acid-mediated in vitro collagen synthesis was measured using picrosirius red staining. Collagen synthesis was significantly increased in HDFs treated with either ADSC-CM or ADSC-EVs (Figure 4). These data suggested that ADSC-CM and ADSC-EVs significantly enhanced collagen synthesis in HDFs.

### 2.5. Gene Expression Differences in Samples Treated with ADSC-CM or ADSC-EVs

**Figure 5:**
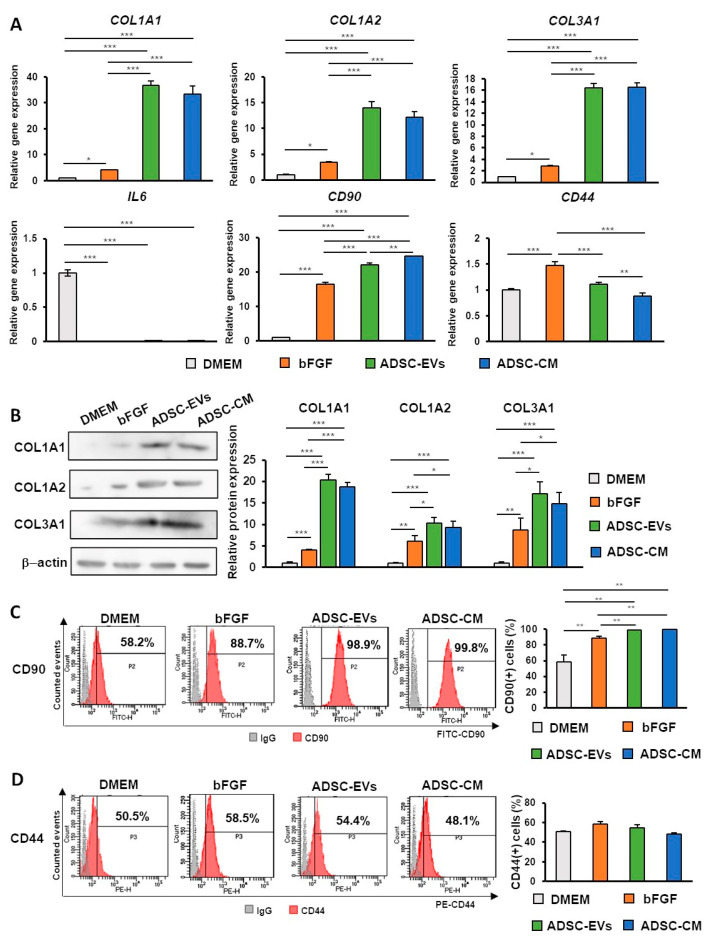
Analysis of wound healing in in vitro radiation-induced injury skin models containing HDFs treated with DMEM, bFGF, ADSC-EVs, or ADSC-CM. (**A**) Gene expression analysis of HDFs treated with DMEM, bFGF, ADSC-EVs, or ADSC-CM examined using qPCR. (**B**) Protein expression of collagen in HDFs treated with DMEM, bFGF, ADSC-EVs, or ADSC-CM examined using Western blotting. (**C**) Protein expression of CD90 in HDFs treated with DMEM, bFGF, ADSC-EVs, or ADSC-CM examined using flow cytometry. (**D**) Protein expression of CD44 in HDFs treated with DMEM, bFGF, ADSC-EVs, or ADSC-CM examined using flow cytometry. (* *p* < 0.05, ** *p* < 0.01, *** *p* < 0.001) (*n* = 3).

Firstly, we focused on evaluating the differences in gene expression between samples. The expression of *COL1A1*, *COL1A2*, and *COL3A1* was significantly increased in the groups treated with ADSC-CM or ADSC-EVs compared with the control and current clinical treatment groups. We also compared the gene expression of *CD44* hyaluronic acid receptors between different experimental groups. *CD44* expression was not significantly different in the control group. Furthermore, we assessed the differences in the inflammatory marker *IL6*. Compared with the control group, *IL6* expression was significantly decreased. In addition, the gene expression differences in *CD90*, a cellular marker for MSCs, were evaluated. *CD90* expression was significantly increased compared with that in the control group (Figure 5A). Next, the protein expression of collagen was compared between the control group and treatment groups using Western blotting. Inconsistent with the gene expression results, the protein expression of COL1A1, COL1A2, and COL3A1 was significantly increased in the groups treated with ADSC-CM or ADSC-EVs compared with the control and current clinical treatment groups (Figure 5B). In addition, the protein expression of CD90 and CD44 was examined using flow cytometry. The results showed that in comparison with the control group, the percentage of cells positive with CD90 was significantly increased in the treatment groups (Figure 5C), while no significant difference in CD44-positive cell percentage was observed between the groups (Figure 5D). Taken together, these data suggested that ADSC-CM and ADSC-EVs can promote radiation-induced skin injury by inducing collagen synthesis and reducing inflammation with better results than the current clinical therapeutic agent bFGF.

## 3. Discussion

It is well known that MSCs have anti-inflammatory and immunomodulatory properties due to the paracrine action of their secreted proteins, which can accelerate skin wound healing and collagen deposition and ameliorate skin damage [28]. However, the potential effect of ADSC-CM and ADSC-EVs on radiation-induced skin injury and the exact mechanism of this effect remains unknown. In this study, we constructed an in vitro radiation-induced skin injury model with HDFs and evaluated the effect of ADSC-CM and ADSC-EVs on radiation-induced skin injury. Our results showed that ADSC-EVs promoted the proliferation of HDFs in a dose-dependent manner. Furthermore, ADSC-CM and ADSC-EVs accelerated the in vitro wound healing caused by radiation exposure by modulating inflammation, reducing HDF apoptosis, and promoting ECM deposition.

HDFs are the most prevalent cells in the human dermis and one of the most important architects of skin wound healing. During the wound-healing process, fibroblasts produce the majority of the ECM components, and simultaneously, these molecules alter the function of fibroblasts [29,30]. The ECM component plays an important role in each stage of the wound-healing process [31]. On the one hand, ECM components relate the biomechanics structural aspects of the process in question, and they form “scaffolds” (temporary matrix, granulation tissue, and scars), which are indispensable in the repair process, thus providing matrix structural integrity during each stage of the healing process [32,33,34,35]. On the other hand, the role of ECM components is also relevant to the role of the healing process, as biochemical mediators, multiple cytokines, and growth factors fulfill a signal transduction function in this dynamic, interactive sequence of biological reactions [36,37,38,39,40]. The latter function involves stimulating cell adhesion and migration during the healing process, as well as mediating interactions among cells, between cells and the matrix, or among ECM proteins [32,33,37,41]. The ECM component is also a reservoir and regulator of the action of cytokines and growth factors, thus modulating wound repair activity [32,36,42,43,44]. Therefore, we established an in vitro model with fibroblasts for our study.

In 2007, Rigotti and colleagues demonstrated for the first time that fat grafting resulted in significant symptomatic improvement in 20 patients with radiation-induced fibrosis after RT for breast cancer [45]. Since this discovery, an increasing number of surgeons have begun using autologous fat grafting to reconstruct previously irradiated tissue [46,47,48]. Although the mechanisms by which autologous fat grafting promotes these beneficial clinical outcomes remain to be elucidated, it is believed that ADSCs in adipose tissue play a major role. Over the past few years, cellular therapies have entered the playing field as possible therapies. ADSC and ADSC-CM have been used in regenerative medicine for the treatment of various diseases [49,50]. Many studies have shown that MSC-EVs have similar biological functions to MSCs themselves [25]. ADSC-EVs also enhance wound healing by modulating inflammatory responses, accelerating fibroblast proliferation, and activating collagen and elastin synthesis in fibroblasts [26,27]. In addition, ADSC-EVs may offer specific advantages for patient safety over whole-cell-based therapies, such as a lower propensity to trigger innate and adaptive immune responses and an inability to directly form tumors [51]. As expected, ADSC-CM as well as ADSC-EVs demonstrated the ability to promote radiation-induced skin injury healing in this study.

Wound healing is a complex and dynamic process that consists of four major overlapping phases: hemostasis, inflammation, proliferation, and remodeling. Excessive inflammation can lead to delayed or even no wound healing [42]. Fibroblasts are strongly involved in the latter three stages [52]. MSCs secrete a variety of growth factors and cytokines that can modulate the response of neutrophils, macrophages, and lymphocytes. It has been shown that MSCs control the Th1–Th2 cytokine balance, triggering the production of anti-inflammatory cytokines such as IL-4 and IL-10, reducing the production of pro-inflammatory cytokines such as IL-6, and exerting an inhibitory effect on natural killer cell activity and cytotoxicity [53]. In our study, the ADSC-CM and ADSC-EV groups showed decreased expression of the pro-inflammatory marker IL-6, suggesting that ADSC-CM and ADSC-EVs could reduce inflammation in radiation-induced skin injury.

The proliferative phase of wound healing is characterized by extensive fibroblast proliferation and ECM deposition, providing suitable conditions for re-epithelialization [42]. As mentioned previously, MSC treatment enhances fibroblast survival and migration and increases ECM deposition by fibroblasts, which in turn enhances healing [22]. MSC-EVs also lead to collagen deposition, which promotes wound healing [54]. In our study, we found that the proliferation of HDFs in the ADSC-EV group was significantly increased in a dose-dependent manner, the apoptosis of HDFs was significantly decreased, and the total amount of collagen synthesis from day 0 to day 14 in the ADSC-CM and ADSC-EV groups was significantly increased, while *CD90*, a marker of MSCs, was significantly increased. The expression of the *CD44* hyaluronic acid receptor was not significantly increased compared with the control group. However, *CD44* expression in the ADSC-EV group showed a tendency to increase and was significantly increased in the ADSC-EV group compared with the ADSC-CM group. Hence, ADSC-EVs might have a somewhat better effect than ADSC-CM. The expression of collagen, including COL1A1, COL1A2, and COL3A1 was also significantly increased on day 14 in both gene and protein levels. These data suggested that ADSC-EVs significantly promoted fibroblast proliferation, while ADSC-CM as well as ADSC-EVs significantly promoted ECM deposition and reduced HDF apoptosis, thereby enhancing wound healing in vitro. 

This study has several limitations. First, the in vitro model in this study was simple, containing only fibroblasts, and was insufficient to represent the effects in terms of the immune response. Furthermore, because it lacked epidermal-related components, it was inadequate in reproducing complete biological skin tissue in vivo. Second, details of the mechanism of action of the effects of ADSC-CM and ADSC-EVs on HDFs in this model are unknown. Therefore, further studies using innovative in vitro and in vivo models of radiation-induced skin injury are necessary. 

## 4. Materials and Methods

### 4.1. Patients

All human tissues, including skin and adipose tissue, were obtained from patients (mean age, 39 ± 13.4 years; age range, 25–57 years; mean body mass index, 22.4 ± 4.1; *n* = 3) who underwent surgical excision at the University of Tsukuba Hospital, Tsukuba, Japan. This study was approved by the ethics committee of the University of Tsukuba Hospital. Approval was obtained from the Tsukuba Clinical Research and Development Organization (T-CReDO; protocol number: R03-263). Written informed consent was obtained from all patients to provide human tissues for research purposes.

### 4.2. Isolation and Culture of ADSCs and HDFs

HDFs were established from surgical specimens using the explant method, as described previously [55]. Briefly, fresh skin tissues obtained during surgical excision were washed three times in phosphate-buffered saline (PBS; FUJIFILM Wako Pure Chemical Corporation, Osaka, Japan). The specimens were then dissected free of fat and epidermis, minced into approximately 1 cm pieces, and attached to a 100 mm cell culture dish that was specially treated for cell culture (TPP; BMBio, Tokyo, Japan). Cells were grown in Dulbecco’s modified Eagle’s medium (DMEM; Thermo Fisher Scientific Inc., Waltham, MA, USA) supplemented with 10% fetal bovine serum (FBS; Thermo Fisher Scientific) and 1% penicillin–streptomycin–amphotericin B suspension (P/S/AB) (FUJIFILM Wako Pure Chemical Corporation, Osaka, Japan) for the primary culture, and the medium was changed every day. The tissues were removed after 7 days of incubation at 37 °C, 5% CO_2_, and 100% humidity. Cells were subcultured until they reached over 80% confluency. After two passages, the cells were switched to DMEM supplemented with 10% FBS and 1% penicillin–streptomycin (P/S) solution (FUJIFILM). HDFs that were passaged 5–6 times were used in the present study.

Isolation and culture of human ADSCs were performed as previously described [56,57,58]. Briefly, adipose tissue was harvested from patients who underwent surgery and digested with collagenase type I (Worthington, Lakewood, NJ, USA) for 30 min in an Isothermal Shaker Water Circulator (Isothermal Shaker Water Circulator T-3S; THOMAS KAGAKU Co., Ltd., Tokyo, Japan) at 37.5 °C. After digestion, the adipocyte suspension was sequentially filtered through 100 µm and 40 µm mesh cell strainers and centrifuged at 300× *g* for 5 min. The cell pellet at the bottom was then resuspended in PBS and filtered through a 100 µm mesh cell strainer. After further centrifugation at 300× *g* for 5 min, the cell pellet was resuspended and cultured in DMEM supplemented with 10% FBS and 1% P/S/AB at 37 °C with 5% CO_2_. ADSCs were subcultured until they reached over 80% confluency. After two passages, the cells were switched to DMEM supplemented with 10% FBS and 1% P/S solution, and cells at passage 5 were used in the present study.

### 4.3. Preparation of ADSC-CM

ADSC-CM was prepared as described previously [57]. Briefly, when ADSCs at passage 5 had reached over 80% confluency, the waste culture medium was removed, and the cells were washed twice in PBS. Subsequently, fresh, serum-free DMEM was added to the cells. The cells were then incubated at 37 °C in a humidified atmosphere containing 5% CO_2_ for 48 h. After 48 h of incubation, the medium was collected and filtered with a 0.22 µm filter. The supernatant obtained after the cell components were filtered was used as the ADSC-CM. FBS was then added to achieve a concentration of 10% when used for experiments.

### 4.4. Isolation and Collection of ADSC-EVs

The EV-enriched ADSC-CM was filtered through a 0.22 µm syringe filter, and ADSC-EVs were extracted using PureExo (101Bio, Palo Alto, CA, USA) according to the manufacturer’s protocol. Briefly, 20 mL of collected ADSC-CM was centrifuged at 3000× *g* for 15 min to remove dead cells and cellular debris. An ADSC-EV extraction reagent mixture was prepared, and the reagent mixture was added to ADSC-CM and mixed thoroughly; then, the pelleted ADSC-EVs were suspended in 200 µL PBS.

### 4.5. Flow Cytometry

To characterize the phenotype of ADSCs, the surface markers were determined using flow cytometry, as previously described [59]. After five passages, the ADSCs were harvested and counted. Approximately 1 × 10^5^ ADSCs were added to 200 µL PBS containing 3% FBS and incubated with antibodies for 30 min at 4 °C in the dark, including FITC-labeled anti-CD90 (561969; BD Biosciences, Franklin Lakes, NJ, USA), PE-labeled anti-CD73 (561014; BD Biosciences), PE-labeled anti-CD105 (560839; BD Biosciences), PE-labeled anti-CD34 (560941; BD Biosciences), FITC-labeled anti-CD31 (303104; Biolegend, San Diego, CA, USA), and FITC-labeled anti-CD45 (304006; Biolegend). The isotype controls included FITC-labeled anti-IgG1 (555748; BD Biosciences) and PE-labeled anti-IgG1 (555749; BD Biosciences). Then, the cells were washed with cold PBS and resuspended in 300 µL PBS containing 3% FBS and analyzed using a flow cytometer (LSRFortessa™ X-20; BD Bioscience). The cells were gated based on their forward and side scatter properties to remove the debris and dead cells at a lower level of forward scatter. Data were recorded on 10,000 large cell events for each group. Populations of cells positive with specific markers were gated with both unstained cells and isotype control-stained cells. 

To examine the expression of CD90 and CD44, HDFs were stained with FITC-labeled anti-CD90 (561969; BD Biosciences) and PE-labeled anti-CD44 (338808; Biolegend). The experiments were performed in triplicate, and the average percentage of cells positive with CD90 or CD44 was calculated.

### 4.6. Western Blotting

To examine EV markers, total proteins were extracted from cell pellets or ADSC-EVs using RIPA lysis buffer (Thermo Fisher Scientific). SDS-polyacrylamide gel electrophoresis was performed with 50 µg protein and then transferred to a PVDF membrane (Merck Millipore, Burlington, MS, USA). After blocking with 2% bovine serum albumin (Thermo Fisher Scientific), the membranes were incubated with primary antibodies, including rabbit anti-CD63 (GTX17441; GeneTex, Irvine, CA, USA), rabbit anti-TSG101 (GTX118736; GeneTex), and rabbit apolipoprotein A1 (APOA1, GTX40453; GeneTex) at 1:1000 dilution at 4 °C overnight. The membranes were then washed and incubated with HRP-conjugated goat anti-rabbit IgG (Invitrogen) secondary antibody at a dilution of 1:10,000 for 2 h at room temperature, followed by incubation with chemiluminescence HRP substrate (EMD Millipore, Burlington, MA, USA) for 1 min. The protein expression was detected using an Image Quant LAS 4000 System (GE Healthcare, Chicago, IL, USA).

To examine the expression of collagen, total proteins were extracted from cell pellets using RIPA lysis buffer (Thermo Fisher Scientific). An amount of 20 µg protein was used for SDS-polyacrylamide gel electrophoresis followed by membrane transferring (Merck Millipore). After blocking with 2% bovine serum albumin (Thermo Fisher Scientific), the membranes were incubated with primary antibodies, including rabbit anti-COL1A1 (GTX82720; GeneTex), rabbit anti-COL1A2 (GTX102996; GeneTex), rabbit anti-COL3A1 (30565; Cell Signaling Technology), and rabbit anti-β-actin (GTX109639; GeneTex), at 1:1000 dilution at 4 °C overnight. The following steps were performed as above. The expression of the protein was quantification using ImageJ (1.53e, Java 1.8.0_172, NIH, Bethesda, MD, USA).

### 4.7. Radiation Conditions and Evaluation of the In Vitro Model

The distance between the radiation source and the in vitro model placed on a turntable was 45 cm. The X-ray apparatus was an MBR-1505R-3 (Hitachi Medical Corporation, Tokyo, Japan) and was operated at 150 kV and 20 mA with 0.5 mm AL and 0.1 mm Cu filtration. Each experimental group reagent was added 1 h before radiation exposure. The radiation dose was as previously described [60]. Radiation was administered as a single dose of 1 Gy per minute over 20 min (total dose of 20 Gy). The radiation-negative group was covered with a 5 mm thick lead plate.

The expression of collagen genes *COL1A1*, *COL1A2*, and *COL3A1*, the inflammatory marker *IL6*, and the stem cell marker *CD90* were compared using qPCR to evaluate the radiation-induced skin injury model established with HDFs. The expression of related genes was determined on day 14 of the culture. Total RNA was isolated from the cells using an RNeasy Plus Mini Kit (Qiagen, Hilden, Germany) according to the manufacturer’s instructions. Subsequently, 0.25 µg total RNA was reverse transcribed into cDNA using a SuperScript VILO cDNA synthesis kit (Invitrogen) according to the manufacturer’s instructions. TaqMan-based qPCR was performed with the following primer/probe sets (Applied Biosystems, San Francisco, CA, USA): *COL1A1* (Hs00164004_m1), *COL1A2* (Hs01028956_m1), and *COL3A1* (Hs00943809_m1). *GADPH* was used as an internal control. The statistical analysis was performed with a two-sample *t*-test. Z scores were utilized to identify outliers that had scores with absolute values of 1.5 or greater for each Ct value in the statistical evaluation of qPCR results [61,62,63].

### 4.8. Cell Proliferation Assay

HDFs were plated in 96-well microplates (TPP; BMBio, Tokyo, Japan) at a density of 2000 cells/well and co-cultured with varying concentrations of ADSC-EVs (25, 50, and 100 µg/mL) or an equal volume of PBS. Cell proliferation was analyzed at 24, 48, and 72 h after ADSC-EV treatment. Cell proliferation was assayed using WST-1 cell proliferation reagent (Roche Diagnostics, Indianapolis, IN, USA) according to the manufacturer’s recommendations, and the optical density (OD) was measured at 450 nm using a microplate reader (Varioskan LUX, Thermo Fisher Scientific) with a reference wavelength of 600 nm to determine the optimal concentration of ADSC-EVs (*n* = 4). Similarly, HDFs were seeded at a density of 2000 cells/well in 96-well microplates (TPP; BMBio, Tokyo, Japan), and each experimental group of reagents was added separately to analyze cell proliferation within 7 days after treatment for each experimental group of reagents. Cell proliferation was also assayed using WST-1 (Roche Diagnostics), and the OD was measured at 450 nm using a microplate reader (Varioskan LUX, Thermo Fisher Scientific) with a reference wavelength of 600 nm (*n* = 5).

### 4.9. TUNEL Assay

Apoptosis in cultures was evaluated using the DeadEnd™ Fluorometric TUNEL System (Promega, Madison, WI, USA). HDFs treated with DMEM, bFGF, ADSC-CM, or ADSC-EVs for 48 h were fixed with 4% paraformaldehyde and then processed in accordance with the manufacturer’s instructions. The fluorescence of apoptotic cells was detected using a Keyence microscope (BZ-X710). Nuclei were counterstained with ProLongTM Gold antifade reagent with DAPI (Invitrogen, Eugene, OR, USA). The number of apoptotic cells was counted manually under an inverted fluorescence microscope (BZ-X710) as previously described [64]. The statistical analysis was performed using a one-way analysis of variance (ANOVA) followed by Tukey’s test.

### 4.10. Collagen Synthesis by HDFs

A collagen synthesis experiment was performed as described previously [61,65]. After HDFs were seeded in 24-well plates and cultured to 100% confluency, bFGF, ADSC-EVs, and ADSC-CM were added with 1000 µM L-ascorbic acid 2-phosphate (Sigma–Aldrich) supplementation to evaluate collagen synthesis. The control group was cultured in DMEM supplemented with 10% Exosome-Depleted FBS (Thermo Fisher Scientific) and 1% P/S. Picrosirius red staining was performed using a commercially available kit (24901; Polysciences, Inc., Warrington, PA, USA) on day 14, according to the manufacturer’s protocol. Images were acquired with a Keyence microscope (BZ-X710). Collagen deposition was quantified by analyzing OD at 550 nm using a microplate reader (Varioskan LUX, Thermo Fisher Scientific). The statistical analysis was performed using a one-way ANOVA followed by Tukey’s test.

### 4.11. qPCR Analysis

The expression of related genes was determined on day 14 of the culture. Total RNA was isolated from the cells using a RNeasy Plus Mini Kit (Qiagen, Hilden, Germany) according to the manufacturer’s instructions. Subsequently, 0.25 µg total RNA was reverse transcribed into cDNA using a SuperScript VILO cDNA synthesis kit (Invitrogen) according to the manufacturer’s instructions. TaqMan-based qPCR was performed with the following primer/probe sets (Applied Biosystems): *COL1A1* (Hs00164004_m1), *COL1A2* (Hs01028956_m1), *COL3A1* (Hs00943809_m1), *IL6* (Hs00174131_m1), *CD44* (Hs01075864_m1), and *CD90* (Hs06633377_s1). *GADPH* was used as an internal control. The statistical analysis was performed using a one-way ANOVA followed by Tukey’s test.

### 4.12. Statistical Analysis

Data are represented as mean ± standard deviation (SD). Two-sample *t*-tests were used for two independent experimental samples, and a one-way ANOVA followed by Tukey’s test or Dunnett’s test was used for three or more independent experimental samples unless specifically indicated. All statistical analyses were performed using EZR (1.55, Saitama Medical Center, Jichi Medical University, Saitama, Japan), which is a graphical user interface for R (4.1.2, The R Foundation for Statistical Computing, Vienna, Austria) [66]. Statistical significance was set at *p* < 0.05. Error bars indicate SD.

## 5. Conclusions

We demonstrated that ADSC-CM plays a beneficial role in healing by reducing inflammation and HDF apoptosis and promoting the deposition of ECM in our simple model of radiation-induced skin injury. Similarly, ADSC-EVs play a beneficial role in healing by reducing inflammation, accelerating the proliferation of fibroblasts, reducing HDF apoptosis, and promoting the deposition of ECM in our model. ADSC-CM and ADSC-EVs may be a promising therapeutic strategy for radiation-induced skin injury.

## Data Availability

The raw data supporting the conclusions of this study will be made available by the authors without undue reservation.

## Supplementary Materials

The following supporting information can be downloaded at: https://www.mdpi.com/article/10.3390/ijms242417214/s1.
