# Supplementary figures and images for: Therapeutic Potential of Adipose-Derived Stem Cell-Conditioned Medium and Extracellular Vesicles in an In Vitro Radiation-Induced Skin Injury Model
Source: Int J Mol Sci. 2023 Dec 7;24(24):17214. doi: 10.3390/ijms242417214 (PMC10743562; doi:10.3390/ijms242417214)

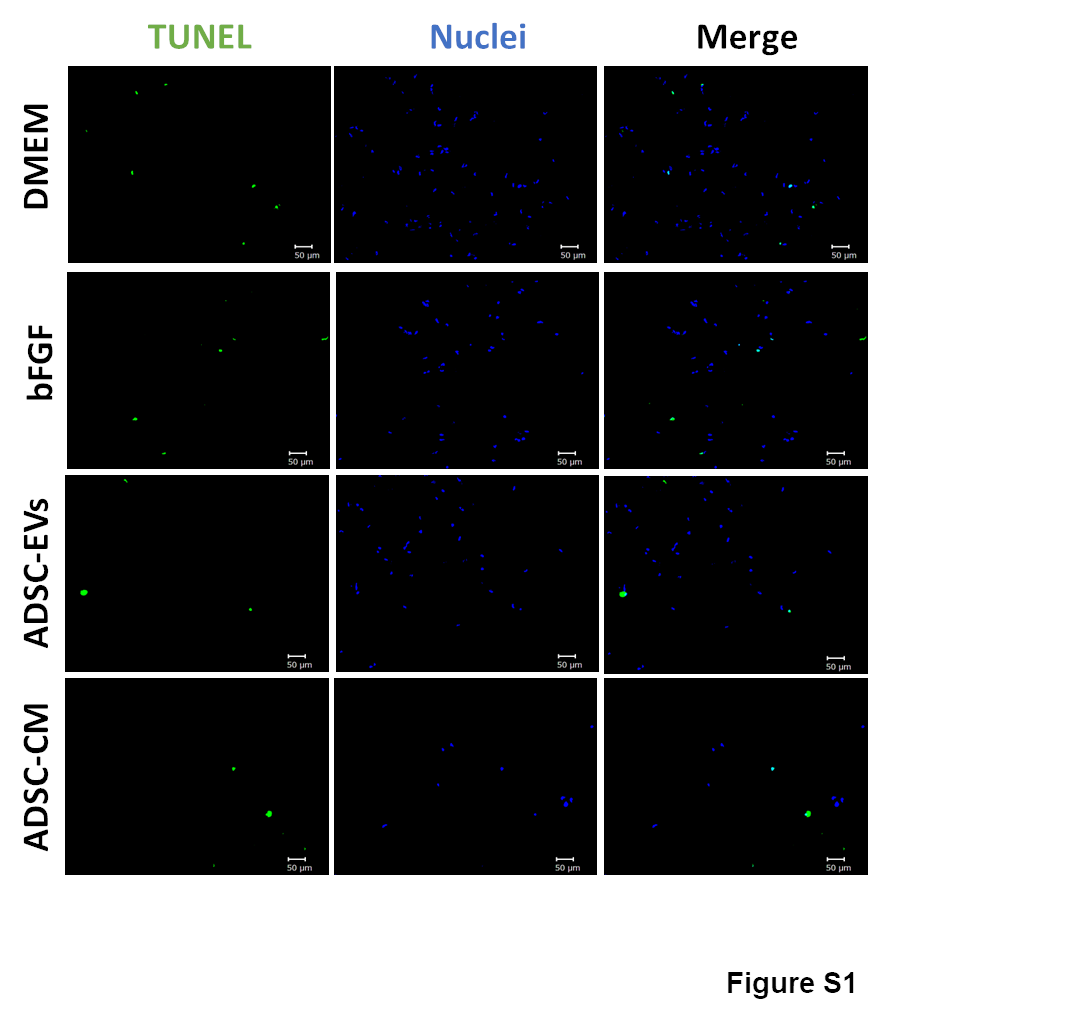

Supplement: Supplementary file 1 [file ijms-24-17214-s001.zip › ijms-2613059-supplementary.png]
